# The Governor’s Appointments

**Published:** 1902-10

**Authors:** 


### TEXAS MEDICAL JOURNAL,

AUSTIN, TEXAS.

A MONTHLY JOURNAL OF MEDICINE AND SURGERY.

EDITED AND PUBLISHED BY

F. E. DANIEL, M. D.

ASSOCIATE EDITOR:

WITTEN BOOTH RUSS, M. D.,

San Antonio, Texas.

Published Monthly at Austin, Texas. Subscription price $1.00 a year in advance.

Eastern Representative: John Guy Monihan, St. Paul Building, 220 Broadway, New York City.

Official organ of the the West Texas Medical Association, the Houston District Medical Association, the Austin District Medical Society, the Brazos Valley Medical Association, the Galveston County Medical Society, and several others.

### THE GOVERNOR'S APPOINTMENTS.

**Dr. Tabor for State Health Officer.**

With the change of the administration in January, it will be in order for the Governor-elect to appoint officers to all the eleemosynary institutions, a State Health Officer and the several quarantine officers. Governor Sayers says that when he went into office wherever he found a good man he retained him, reappointed him, and I believe that he did, so far as he knew, and the policy is a good one—the only correct one. This is not saying, however, that he did not make some very bad appointments, notably three, which he has had abundant reason to regret. The majority of the others have been efficient, and have reflected credit on his administration. This is especially true of the superintendents and assistants of the several insane asylums and the State Health Officer and the quarantine officers. Especially fortunate, in my opinion, was his selection of Dr. George R. Tabor to succeed Blunt (resigned) as State Health Officer. He could not have found in Texas a better man for the position amongst those eligible and who could have accepted it. Dr. Tabor's administration of the quarantine has been characterized by zeal, intelligence, efficiency and thoroughness, and has given universal satisfaction. The medical profession of the State—with whom Dr. Tabor is very popular, and stands A1 as an ethical physician, and courteous gentleman—are especially proud of him and his record. It has reflected credit on them, and they will endorse him most cordially, and on the Governor and on the State of Texas. He has harmonized the commercial interest, heretofore always at war with the quarantine, and the *business men* will endorse him. It is earnestly hoped and believed that Mr. Lanham, who is a broad and liberal statesman, will see the wisdom and the justness and the advantage to the public health and to the unfortunates in our big charity institutions of carrying out the policy inaugurated by Mr. Sayers, by reappointing all of the officers enumerated above. Especially would it be wise, judicious, just; and from every point of view desirable, to retain in the service of the State at the head of the health department the man who has given such signal satisfaction and evidence of a rare administrative and executive ability, a knowledge of sanitary science, and the necessity of progress and reform. It would be unpardonable, the veriest folly, and a mistake to appoint a new and inexperienced man to one of these positions. Neither political service, "pull," nor geographical considerations should have any weight in the selection; but fitness, qualification, integrity of character, professional standing and the *record* should be the paramount consideration.

Dr. Tabor's report to the Governor is now in press. It is in the nature of a stunner. I have been permitted access to the advance sheets and I reproduce, with the doctor's permission, the following extracts. I regret that I can not give an extended review of the report, for it is full of interest on many points, and shows not only a grasp of the situation, but a comprehensive knowledge of what is needed to strengthen, enlarge and perfect the health department. I will do so later, when the report is issued. Dr. Tabor "got onto" the Jones steal and the Tackaberry steal (some $10,000) before he had gotten well warm in his seat, and put the officers on to it. It had been going on under Blunt's nose (or eyes) several months.

Of the amount appropriated for all purposes (quarantine department) a smaller appropriation than that heretofore made—

supplemented by the receipts from the quarantine station—Dr Tabor put twenty-six thousand back into the treasury! This is unprecedented. It shows—well, we won't say what it shows—except that he is par excellence the man for the place. The following figures speak for themselves.

(From advance sheets State Health Officer's report, 1901-2.)

APPROPRIATIONS FOR YEAR ENDING AUGUST 31, 1902.

| | | |
|---|---|---|
| August 31, 1902—Appropriation for salaries | $ 29,650 00 | |
| August 31, 1902—Expenditures for salaries | 27,995 07 | |
| September 1, 1902—Balance unexpended.. | | $ 1,654 93 |
| August 31, 1902—Appropriation for maintenance | $ 12,000 00 | |
| August 31, 1902—Expenditures for maintenance | 10,099 63 | |
| September 1, 1902—Balance unexpended.. | | 1,900 37 |
| August 31, 1902—Appropriation fumigating vessel | $ 45,000 00 | |
| August 31, 1902—Amount contract, fumigating vessel | 37,250 00 | |
| September 1, 1902—Balance unexpended.. | | 7,750 00 |
| September 1, 1902—Total balance appropriation unexpended | | $ 11,305 30 |
| To this balance should be added........ | | $ 15,084 65 |

That is the amount collected for quarantine fees for year ending August 31, 1902, and which has formerly been used by this department.

*This makes a total balance turned back into the treasury of $26,389.95.* This does not include the $15,000 appropriated for quarantine officer's residence at Galveston, none of which amount has been spent.

You will pardon me for calling your Excellency's attention to this unprecedented large amount of money reverting to the treasury from this department.

It will be seen that Dr. Tabor recommends the creation of a State Board of Health. He is a member of the Texas Medical Association's committee on State Board of Health. If the Legislature will make the same appropriation for quarantine purposes as heretofore, the surplus will pay all the additional expense of a State Board and no further appropriation will be required or asked for in the bill.

It will be seen, also, that Dr. Tabor recommends that authority be given the health department to compel railroads to disinfect their cars, and hotels and boarding-houses their rooms, because in the immense number of travelers coming south and especially into Texas there are always persons suffering from consumption and other communicable diseases; and the diseases are spread by persons sleeping in the rooms or cars recently occupied by them. In no intelligent community would a *known infected house* be permitted to be occupied until it had been disinfected. Yet the unsuspecting traveling public are subjected to the deadly contagion of fatal diseases by being put into infected sleepers and rooms of hotels.

Dr. Tabor says:

TUBERCULOSIS.

This dread disease is the most serious we have to contend with and one with which fewer precautionary measures are taken.

Texas is known the world over as nature's sanitarium for consumptives and the result is that our State is continually being infected by such patients from other parts of the world. I believe railway trains and hotels should be required to thoroughly disinfect their coaches and rooms after being occupied by tuberculous patients. * * *

As this disease prevails to such a great extent among poor people of this State—people who live in poverty and filth, making their surroundings a hotbed of infection—at times communicating it to others who desire to render them aid and alleviate their suffering, and as no provision is made to care for them properly, it is my humble judgment that the State government should provide a hospital in some part of her health-giving, mountainous territory where those who are unable to care for themselves should be given an opportunity to recover their health. This is not due only to the individuals who are sick, but it will be a protection to the public to have them removed from their midst. * * *

BUREAU OF VITAL STATISTICS.

A law should be passed requiring all physicians and midwives in the State to report to their local health officers all births and deaths occurring in their practice, a record of which should be kept and reported monthly by the local health officer to this department. Penalties should be prescribed for failure to observe this law.

I am most heartily in favor of the creation of a bureau of vital statistics, which should be under the supervision of the head of this department. The expense necessary for the management of this bureau would be very small, and the benefits to the general public resulting therefrom would be great. * * *

STATE BOARD OF HEALTH.

For many years efforts have been made by the committees appointed by the State Medical Association to have the Legislature authorize the creation of a State Board of Health. I think a board of health organized in a proper manner and given authority to create health laws would be of material benefit to the public. I favor strongly the creation of a State Board of Health with the State Health Officer as ex-officio president of the board and its executive officer.

I do not believe it advisable or expedient for the State Health Officer to be deprived of his present authority (which could be vested in him by a State Board of Health), nor do I believe the present system of coast and border quarantine should be interfered with.

The present system of quarantine, the control of which is vested in the Governor and the State Health Officer, has been in existence in this State since 1879, and it has certainly been a protection to our people. It is eminently satisfactory, and since its organization, my distinguished predecessors, all men of acknowledged ability, and to whom the thanks of our entire people are due, who have, with the advice of the chief executive, administered quarantine, has kept the State free from fatal contagious and infectious diseases, while adjoining States, whose quarantine is controlled by boards of health, have suffered many times by the visitations of that most dreaded scourge to our people, yellow fever; while, by the superior management and untiring efforts of former State Health Officers, our State has been kept free from this disease, with the exception of a few mild cases in Southern Texas in 1897 and 1898, no fatal epidemic of yellow fever having occurred in this State since the organization of the Quarantine Department. I do not think the present system of management of quarantine should be interfered with. * * *

